# SARS-CoV-2 ORF8 Exploits Host miRNA Networks to Rewire Post-Transcriptional Regulation

**DOI:** 10.3390/ijms27188363

**Published:** 2026-09-19

**Authors:** Raúl Fernández-Rodríguez, José M. Suárez-Cárdenas, Antonio Romero-Guillén, Blanca D. López-Ayllón, Fátima Milhano Santos, Ana de Lucas-Rius, Unai Merino-Herrán, Fernando Corrales, María Montoya, Juan J. Garrido, Tránsito García-García

**Affiliations:** 1 Immunogenomics and Molecular Pathogenesis Group, UIC Zoonoses and Emergent Diseases ENZOEM, Department of Genetics, University of Córdoba, 14071 Córdoba, Spain; b42feror@uco.es (R.F.-R.); ge1gapaj@uco.es (J.J.G.); 2 Maimónides Biomedical Research Institute of Córdoba (IMIBIC), 14004 Córdoba, Spain; 3 Viral Immunology Lab, BIC Unit, Molecular Biomedicine Department, Margarita Salas Center for Biological Research (CIB-CSIC), 28040 Madrid, Spain; 4 Biological Defense Area, CBRN Defense Department, General Subdirectorate for Terrestrial Systems—Campus “La Marañosa”, National Institute for Aerospace Technology (INTA), 28330 Madrid, Spain; 5 Functional Proteomics Laboratory, Centro Nacional de Biotecnología, CSIC, Calle Darwin 3, Campus de Cantoblanco, 28049 Madrid, Spain

**Keywords:** SARS-CoV-2, ORF8, microRNAs, post-transcriptional regulation, host–virus interactions, proteomics

## Abstract

Severe acute respiratory syndrome coronavirus 2 (SARS-CoV-2) encodes several accessory proteins that modulate host cellular pathways and contribute to immune evasion and disease severity. Among them, ORF8 has emerged as a key immunomodulatory factor; however, its impact on host post-transcriptional regulatory networks remains poorly defined. In this study, we investigated how ORF8 expression reshapes the microRNA (miRNA) landscape of lung epithelial cells and how these changes translate into functional proteomic alterations. Using A549 cells transduced with ORF8 and control cells, we performed small RNA sequencing to identify differentially expressed miRNAs, followed by integrative analysis with quantitative proteomic data. ORF8 expression induces extensive remodeling of the miRNA profile, with coordinated upregulation and downregulation of miRNAs involved in inflammatory signaling, interferon regulation, apoptosis, autophagy and epithelial homeostasis. Integration of miRNA and proteomic datasets revealed inverse regulatory relationships linking ORF8-associated miRNAs to proteins involved in immune defense, stress responses and epithelial integrity. Selected miRNA alterations were validated by RT–qPCR, and the junctional protein plakoglobin (JUP) was experimentally confirmed as a representative downregulated target. Collectively, our findings identify ORF8 as a factor associated with coordinated miRNA-mediated post-transcriptional reprogramming in lung epithelial cells. This network-based regulatory framework provides new insight into how SARS-CoV-2 accessory proteins may influence host responses and highlights miRNA networks as potential contributors to viral pathogenesis and host immune dysregulation.

## 1. Introduction

Severe acute respiratory syndrome coronavirus 2 (SARS-CoV-2), the causative agent of the COVID-19 pandemic, has exerted an unprecedented impact on global public health and socioeconomic systems. Although SARS-CoV-2 primarily infects the respiratory tract, the clinical spectrum of COVID-19 reflects a complex interplay between viral factors and host cellular responses [1,2]. In addition to its structural and non-structural proteins, SARS-CoV-2 encodes several accessory proteins that modulate host pathways, facilitate immune evasion and contribute to disease severity [3,4].

Among these accessory proteins, ORF8 has emerged as a multifunctional immunomodulatory factor. Multiple studies have demonstrated that ORF8 interferes with major histocompatibility complex class I (MHC-I) presentation by directly binding surface MHC-I molecules and targeting them for lysosomal and autophagic degradation [5]. Furthermore, ORF8 impairs type I interferon (IFN) antiviral responses through a dual mechanism: it directly antagonizes host innate sensing factors to prevent the nuclear translocation and phosphorylation of IRF3, while also dampening the downstream STAT1/STAT2 pathway to suppress interferon-stimulated gene (ISG) transcription [6,7,8], thereby reducing cytotoxic T cell recognition and promoting immune evasion. In addition, ORF8 has been described as a viral cytokine-like protein capable of inducing pro-inflammatory responses, including increased interleukin-6 (IL-6) production, which may contribute to the hyperinflammatory phenotype observed in severe COVID-19 [9,10]. ORF8 has also been shown to interact with components of the endoplasmic reticulum and protein quality control machinery, triggering ER stress-like responses and modulating intracellular signaling pathways [11]. Together, these findings highlight ORF8 as a key determinant of host immune dysregulation during SARS-CoV-2 infection.

MicroRNAs (miRNAs) are small non-coding RNAs that regulate gene expression at the post-transcriptional level by targeting messenger RNAs for degradation or translational repression. miRNAs play essential roles in immune regulation, inflammation, apoptosis, metabolism and epithelial homeostasis [12,13]. Furthermore, recent in silico systems analyses have demonstrated the power of studying miRNA-regulatory networks to understand the complex transcriptional and post-transcriptional control of the host immune response during viral infections, further underscoring the importance of investigating how viral proteins can manipulate these networks [14]. During viral infection, host miRNA expression profiles are frequently altered, reflecting both antiviral defense mechanisms and virus-driven manipulation of host regulatory networks [15]. In the context of SARS-CoV-2 infection, several studies have reported widespread changes in host miRNA expression, with specific miRNA signatures associated with immune dysfunction and tissue damage [16,17]. Moreover, coronaviruses can directly exploit the host miRNA network to promote pathogenesis [18], and miRNA expression patterns have been shown to predict infection severity [19]. Despite this growing body of work, the contribution of individual SARS-CoV-2 accessory proteins to miRNA-mediated post-transcriptional regulation remains poorly understood.

Notably, although ORF8 has been extensively studied at the protein and transcriptional levels, its potential role in reshaping host miRNA networks has received limited attention. Given the central role of miRNAs as integrators of cellular signaling and stress responses, changes in miRNA expression associated with ORF8 could represent an efficient mechanism to coordinately reprogram multiple host pathways. Understanding whether ORF8 influences miRNA-mediated regulation is therefore critical to fully elucidate its contribution to immune evasion, inflammation and epithelial dysfunction during SARS-CoV-2 infection.

In this study, we investigated the impact of ORF8 expression on the miRNA landscape of lung epithelial cells, a primary cellular target of SARS-CoV-2 infection. Using A549 cells transduced with ORF8 and control cells, we performed small RNA sequencing to identify differentially expressed miRNAs associated with ORF8 expression. To assess the functional consequences of these changes, we integrated miRNA profiling with quantitative proteomic data obtained from the same experimental system. This integrative approach enabled the identification of coordinated miRNA–protein regulatory networks and the experimental validation of selected miRNA–target relationships. Our findings provide novel insight into miRNA-mediated post-transcriptional mechanisms underlying ORF8-driven host reprogramming and highlight miRNA networks as an additional layer of SARS-CoV-2–host interaction.

## 2. Results

### 2.1. ORF8 Expression Induces Extensive Remodeling of the Host miRNA Landscape

**Figure 1:**
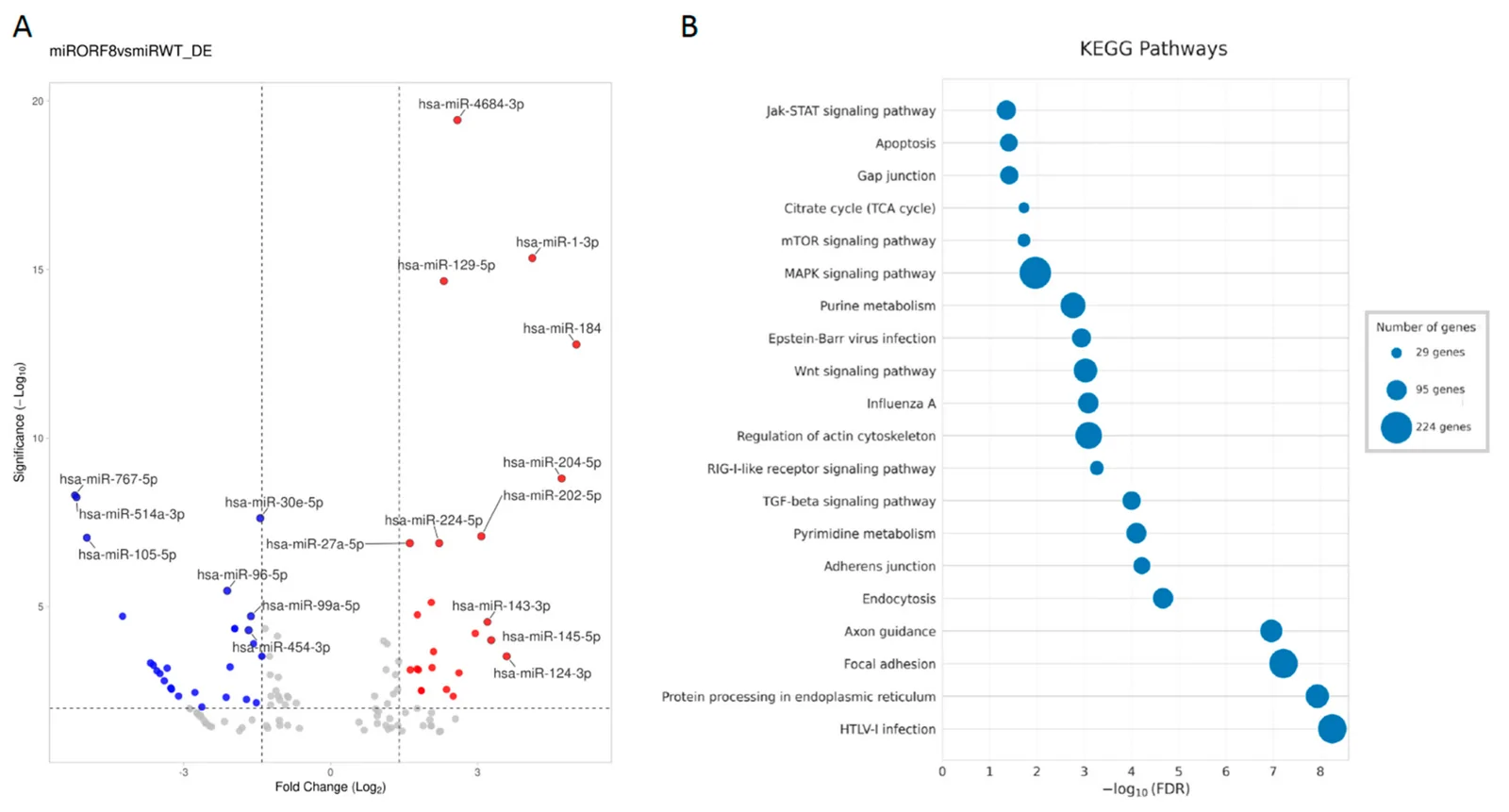
ORF8 expression reshapes the host miRNA landscape. (**A**). Volcano plot showing differentially expressed miRNAs in ORF8-transduced A549 cells compared with control cells. The x-axis represents log2 fold change and the y-axis represents −log10 adjusted *p*-value. Significantly up- and downregulated miRNAs are highlighted. (**B**). Pathway enrichment analysis of predicted miRNA target genes. Dot size represents the number of target genes associated with each pathway.

To investigate the global impact of ORF8 on host post-transcriptional regulation, A549 pulmonary epithelial cells were transduced with lentiviral particles encoding SARS-CoV-2 ORF8 or an empty vector control, followed by selection with puromycin (2 µg/mL) to establish a homogeneous population expressing ORF8. Subsequently, we performed small RNA sequencing in A549 cells expressing ORF8 and compared them with control cells. ORF8 expression and sub-cellular localization in this transduced A549 cell model were confirmed in line with our previously established characterization [20]. Differential expression analysis revealed extensive and statistically robust remodeling of the miRNA landscape upon ORF8 expression (adjusted *p*-value < 0.05; Supplementary Table S1). As shown in the volcano plot (Figure 1A), numerous miRNAs displayed significant differential expression, with several exhibiting large log2 fold changes and high statistical confidence. These results are consistent with previous reports showing that viral proteins can profoundly remodel host miRNA expression to modulate immune responses, apoptosis and cellular metabolism [21,22].

Among the miRNAs analyzed, the most significantly upregulated miRNA was hsa-miR-4684-3p, with a log_2_FC of 2.5897 and a highly significant padj of 3.66 × 10^−20^. Similarly, hsa-miR-1-3p displayed a log_2_FC of 4.1173 (padj = 4.57 × 10^−16^), suggesting a strong induction by ORF8. Other notably upregulated miRNAs, hsa-miR-184 and hsa-miR-129-5p, exhibited a log_2_FC of 5.0154 (padj = 1.67 × 10^−13^) and 2.3122 (padj = 2.21 × 10^−15^), respectively, in ORF8 conditions. hsa-miR-202-5p was also strongly induced in ORF8-transduced cells with a log_2_FC of 3.0748 (padj = 8.19 × 10^−8^). Conversely, several miRNAs were significantly downregulated in the presence of ORF8. hsa-miR-767-5p displayed the most significant downregulation, with a log_2_FC of −5.212 (padj = 4.97 × 10^−9^) and no detectable reads in ORF8-transduced cells compared to control cells. Similarly, hsa-miR-514a-3p was also completely absent in ORF8 conditions (0 reads) compared to control cells, resulting in a log_2_FC of −5.1821 (padj = 5.73 × 10^−9^). hsa-miR-105-5p was also silenced after expression of ORF8, with a log_2_FC of −4.9717 (padj = 9.05 × 10^−8^). Additional downregulated miRNAs included hsa-miR-3176 (log_2_FC = −4.2427, padj = 1.91 × 10^−5^) and hsa-miR-509-3-5p (log_2_FC = −3.6737, padj = 0.00046), with no detectable expression in ORF8 cells compared to control.

The differential expression analysis revealed a balanced regulatory pattern between miRNAs induced and repressed by ORF8. Of the top 30 most significant miRNAs (padj < 0.001), 14 exhibited positive log_2_FC values (upregulation), while 16 showed negative log_2_FC values (downregulation) (Table 1).

### 2.2. Functional Enrichment Analysis Links ORF8-Regulated miRNAs to Inflammatory, Epithelial and Stress-Response Pathways

Based on differential expression analysis (Figure 1A, Table 1), we selected the most significantly up- and downregulated miRNAs to explore their potential impact on key cellular processes. We then examined their known or predicted roles using existing literature and miRNA databases (mirTarBase v9.0 and TarBase v9.0). Pathway enrichment analysis of predicted miRNA target genes revealed significant over-representation of pathways related to endoplasmic reticulum protein processing, cell adhesion, cytoskeletal regulation, apoptosis and immune-related signaling pathways (Figure 1B). Notably, several enriched pathways, including MAPK, mTOR, TGF-β and RIG-I-like receptor signaling, have been previously implicated in coronavirus infection, innate immune regulation and epithelial stress responses [1,23].

Among the upregulated miRNAs, several have been previously associated with inflammatory signaling and cytokine regulation. hsa-miR-1-3p has been reported to regulate cell proliferation and apoptosis in lung epithelial cells, and its induction is consistent with stress-associated cellular responses described in inflammatory contexts [24,25]. Notably, it has also been recently identified as a predictor of disease severity in hospitalized COVID-19 patients [26], further supporting its potential clinical relevance. hsa-miR-129-5p and hsa-miR-224-5p have been linked to cytokine regulation and cell migration, with hsa-miR-129-5p reported to target *IL6* and TNF-α, supporting its involvement in cytokine-associated inflammatory pathways [27]. Additional upregulated miRNAs, including hsa-miR-27a-5p, hsa-miR-1246 and hsa-miR-451a, have been associated with NF-κB signaling and the regulation of *IL-1β* and *IL-6* release, further linking ORF8-associated miRNA changes to inflammatory response pathways [28,29,30].

A second subset of upregulated miRNAs is primarily linked to cell survival and apoptosis-related pathways. hsa-miR-184, hsa-miR-204-5p and hsa-miR-202-5p have been reported to regulate *AKT2*, *FOXO1* and *BCL2*, respectively, consistent with roles in survival and anti-apoptotic signaling [31,32,33]. In addition, hsa-miR-124-3p has been shown to target *STAT3*, NF-κB-associated components and *SNAI2*, linking it to the regulation of antiviral cytokine signaling and epithelial plasticity [34,35]. Collectively, these findings indicate that upregulated miRNAs in ORF8-transduced cells converge on pathways related to inflammation, survival and epithelial regulation.

Several downregulated miRNAs identified in ORF8-transduced cells have been previously implicated in antiviral and interferon-associated responses. hsa-miR-30e-5p, hsa-miR-374b-5p and hsa-miR-454-3p have been reported to enhance type I interferon signaling through the regulation of *SOCS1*, *SOCS3* and components of the PTEN–IRF3 axis during viral infection [36,37,38]. Their reduced expression is therefore consistent with attenuation of interferon-associated regulatory mechanisms.

In addition, other downregulated miRNAs are linked to autophagy, stress responses and epithelial homeostasis. hsa-miR-99a-5p and hsa-miR-514a-3p have been associated with regulation of the mTOR pathway and autophagic processes [39,40], while hsa-miR-96-5p and hsa-miR-455-5p have been reported to target regulators of NF-κB signaling and immune checkpoint molecules such as PD-L1 [41,42]. Furthermore, hsa-miR-374b-5p and hsa-miR-454-3p have been linked to the regulation of junctional and stress-response proteins, including *MAPK1* and *ZEB1*, suggesting a potential impact on epithelial integrity and barrier-associated functions [37,43].

### 2.3. ORF8-Modulated miRNAs Overlap with miRNA Signatures Reported in COVID-19 Patients

**Figure 2:**
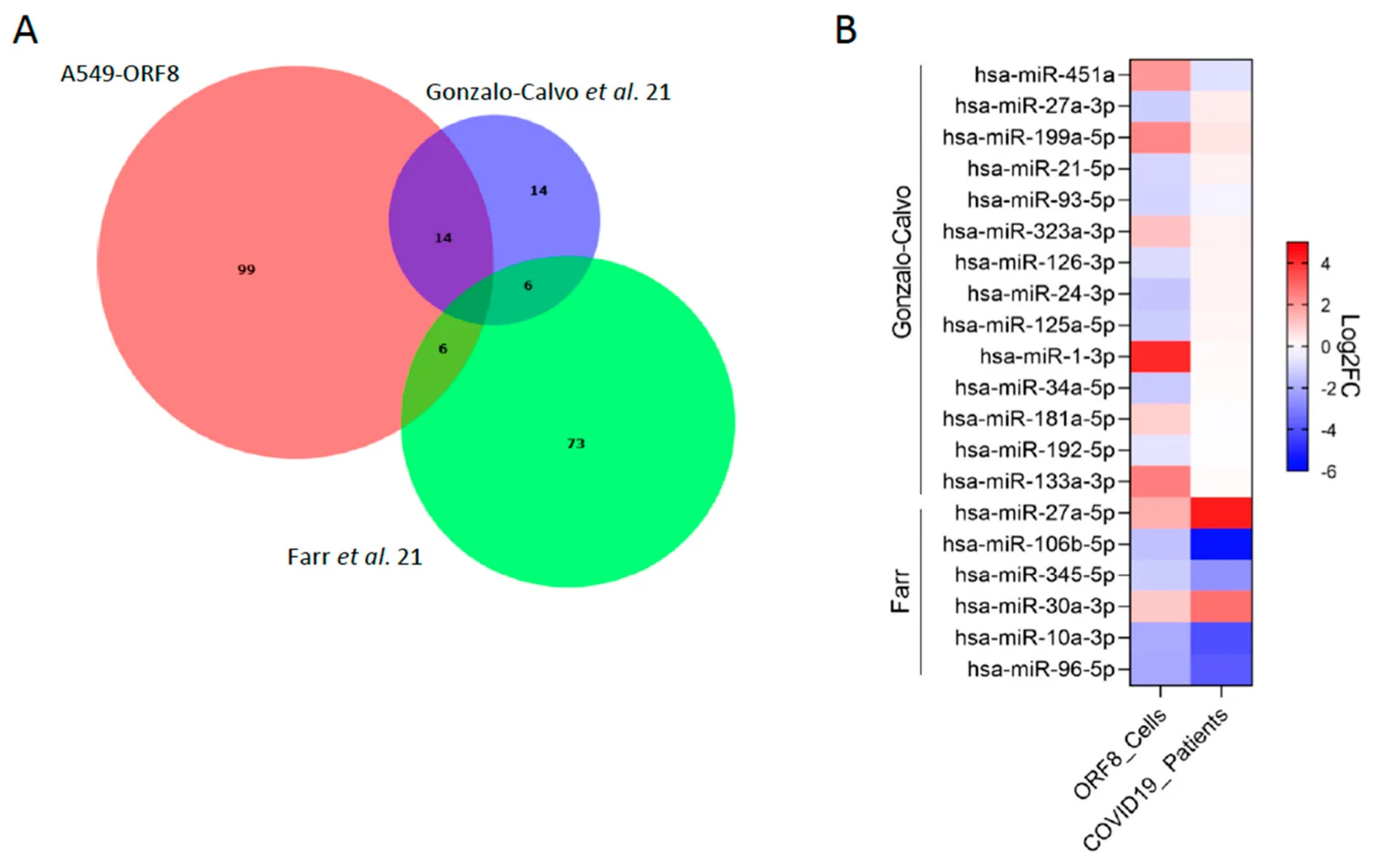
Overlap between ORF8-induced miRNA signatures and miRNAs reported in COVID-19 patients [44,45]. (**A**). Venn diagram showing the intersection between differentially expressed miRNAs identified in ORF8-transduced A549 cells and miRNAs previously reported to be dysregulated in COVID-19 patient samples. (**B**). Heatmap representation of selected overlapping miRNAs, displaying their expression patterns in ORF8-transduced cells and in COVID-19 patient datasets. Values represent log_2_ fold change, with upregulated and downregulated miRNAs indicated by a color scale.

To assess whether the ORF8-induced miRNA profile resembles clinically relevant host responses, we performed an exploratory comparison of our dataset with published miRNA signatures from two benchmark clinical cohorts [44,45]. These well-cited studies were selected as primary references because they provided comprehensive, high-throughput global miRNA profiling in hospitalized COVID-19 patients across a defined spectrum of disease severity. Specifically, the study by de Gonzalo-Calvo et al. analyzed hospitalized COVID-19 patients, including those with severe disease requiring mechanical ventilation [44]. The study by Farr et al. profiled a cohort of patients with a spectrum of disease severity, from mild to critical, also requiring hospitalization [45] (Figure 2A). Notably, several miRNAs were shared between ORF8-transduced cells and individual patient datasets, further supporting a partial convergence between the in vitro ORF8-driven signature and clinically derived miRNA profiles.

To further explore the directionality and magnitude of these changes, we generated a heatmap of selected overlapping miRNAs (Figure 2B). This analysis demonstrated that several miRNAs exhibit concordant regulation between ORF8-transduced cells and COVID-19 patient samples, including the upregulation of hsa-miR-27a-3p and hsa-30a-3p, as well as the downregulation of specific miRNAs such as hsa-miR-96-5p. These patterns are consistent with the reported roles of these miRNAs in regulating inflammatory responses [28,41]. hsa-miR-1-3p, which has been recently reported as a predictor of disease severity in hospitalized COVID-19 patients, was also detected in our dataset with a relatively high fold change, further supporting its potential clinical relevance.

Interestingly, a number of miRNAs identified in our dataset have not been previously associated with COVID-19, suggesting that ORF8 may drive previously unrecognized miRNA regulatory networks.

Together, these findings indicate that ORF8 is sufficient to induce a subset of miRNA changes that have also been reported in COVID-19 patient cohorts. While this does not establish a causal link, it suggests that ORF8 may contribute, alongside other viral and host factors, to clinically observed miRNA profiles. The limited overlap may reflect the complexity of the in vivo environment, which includes contributions from other viral proteins, immune cells, and the host genetic background, factors not present in our controlled in vitro system. Nonetheless, this partial overlap highlights the potential contribution of ORF8 to clinically relevant miRNA signatures associated with SARS-CoV-2 pathogenesis.

### 2.4. Proteomic Alterations Induced by ORF8 Reflect Immune and Stress-Related Reprogramming

**Figure 3:**
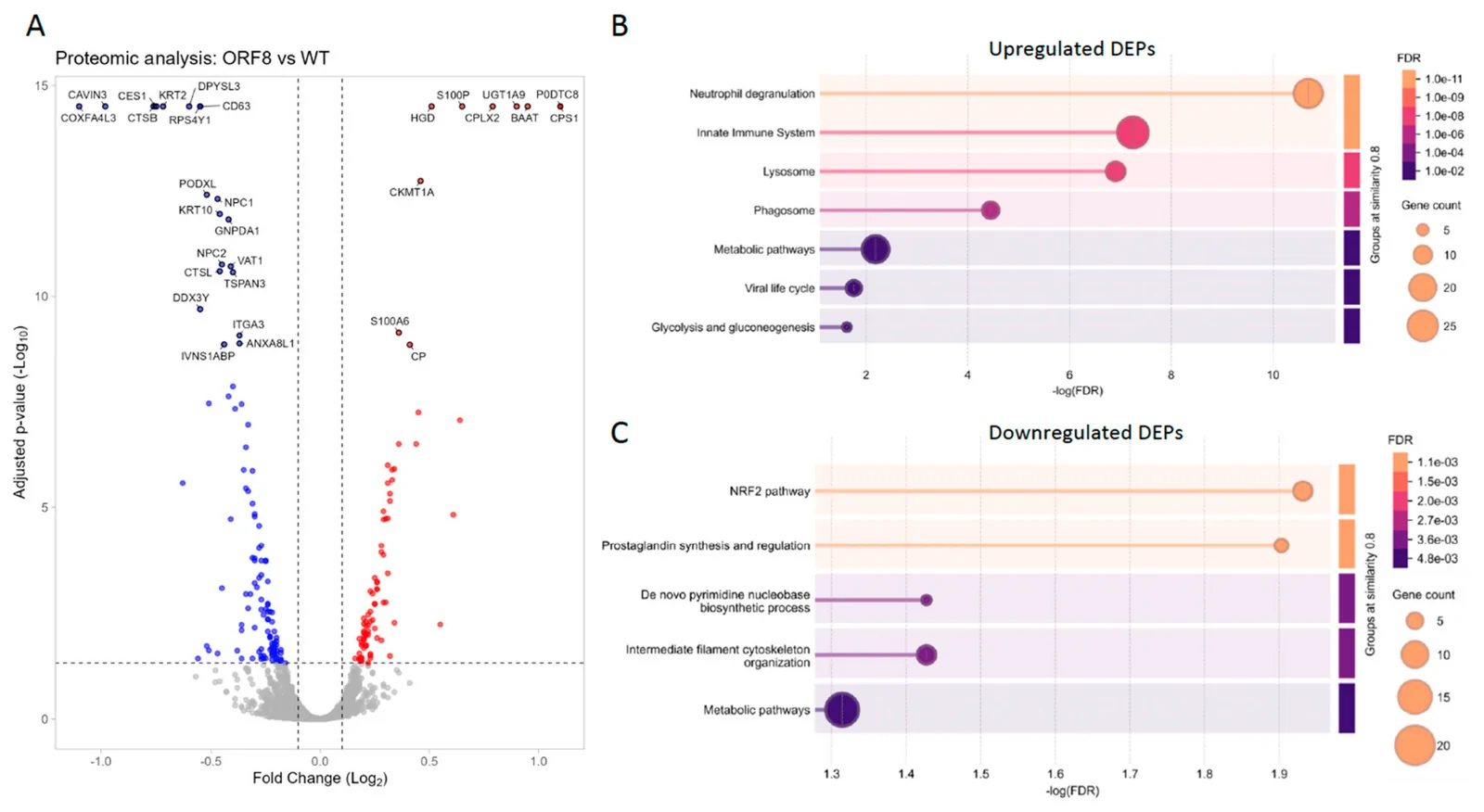
Functional stratification of differentially expressed proteins in ORF8-transduced cells. (**A**). Volcano plot showing differentially expressed proteins (DEPs) in ORF8-transduced A549 cells compared with control cells. The x-axis represents log2 fold change and the y-axis −log10 adjusted *p*-value. Significantly up- and downregulated proteins are highlighted. (**B**). Enriched pathways associated with up-regulated DEPs, highlighting immune defense, lysosomal-related processes, viral life cycle and metabolic pathways. (**C**). Enriched pathways associated with down-regulated DEPs, including metabolic adaptation, stress response pathways and cytoskeleton organization. Dot size represents the number of target genes associated with each pathway.

To determine whether ORF8-induced miRNA remodeling translated into functional protein-level changes, we next analyzed quantitative proteomic data obtained from the same experimental system. Differential protein expression analysis identified a distinct set of significantly upregulated and downregulated proteins in ORF8-expressing cells compared with controls, as illustrated by the volcano plot (Figure 3A). Representative differentially expressed proteins (DEPs) highlight the broad impact of ORF8 on cellular protein abundance.

DEPs were subsequently stratified into upregulated and downregulated groups and subjected to pathway enrichment analysis. Upregulated DEPs were significantly associated with pathways related to immune defense, lysosomal function, viral life cycle and metabolic processes (Figure 3B). These findings are consistent with the reported ability of ORF8 to modulate immune-related pathways and intracellular trafficking, potentially contributing to immune evasion and viral persistence [5]. In contrast, downregulated DEPs were enriched for pathways involved in cytoskeleton organization, cellular stress responses and metabolic adaptation (Figure 3B). Disruption of cytoskeletal dynamics and cellular architecture has been linked to impaired epithelial barrier function and altered immune signaling during SARS-CoV-2 infection [46]. Together, these results indicate that ORF8 induces coordinated protein-level changes consistent with the miRNA-predicted disruption of inflammatory regulation, epithelial structure and metabolic adaptation.

### 2.5. Experimental Validation and Network Integration Identify Coordinated miRNA–Protein Regulatory Modules

**Figure 4:**
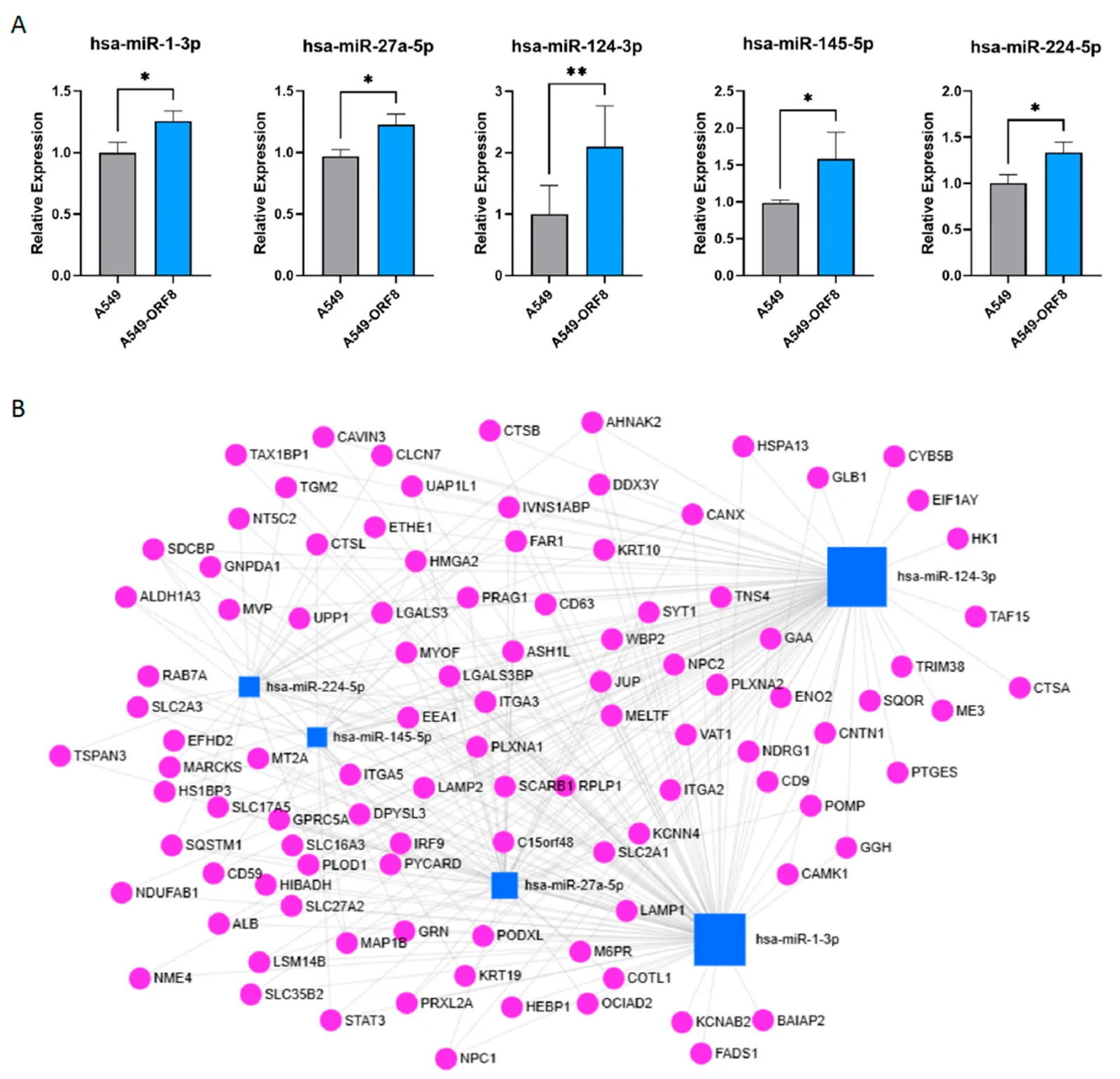
Validation and network-based integration of ORF8-upregulated miRNAs. (**A**). RT–qPCR validation of selected miRNAs significantly upregulated in ORF8-transduced A549 cells compared with control cells. Relative expression levels are shown for hsa-miR-1-3p, hsa-miR-27a-5p, hsa-miR-124-3p, hsa-miR-145-5p and hsa-miR-224-5p. Data are expressed as mean ± SEM from independent 3 experiments and are normalized to endogenous controls. Statistical analysis was performed using an unpaired two-tailed Student’s *t*-test (* *p* < 0.05; ** *p* < 0.01). (**B**). Network representation of ORF8-upregulated miRNAs and their predicted or experimentally supported protein targets. Upregulated miRNAs are shown in blue, and protein targets are shown in pink. Edges represent miRNA–protein interactions identified using integrative miRNA–proteome analysis.

To validate the small RNA sequencing results, a subset of significantly upregulated miRNAs was selected for RT–qPCR validation. These miRNAs were chosen based on fold change, statistical significance and predicted functional relevance. RT–qPCR analysis confirmed significant upregulation of hsa-miR-1-3p, hsa-miR-27a-5p, hsa-miR-124-3p, hsa-miR-145-5p and hsa-miR-224-5p in ORF8-transduced cells compared with control cells (Figure 4A), supporting the robustness of the sequencing data.

To better understand how ORF8-modulated changes in the miRNA landscape translate into functional alterations at the protein level, we integrated miRNA and proteomic datasets. ORF8-upregulated miRNAs were mapped to their predicted or experimentally supported protein targets, generating a miRNA–protein interaction network (Figure 4B). The integration strategy is based on the principle of inverse regulation between miRNAs and their targets: upregulated miRNAs are expected to suppress specific proteins, while downregulated miRNAs may lead to de-repression and increased protein levels. To systematically explore these interactions, we employed miRNet [47], a well-established bioinformatics platform for miRNA–target network construction. Specifically, we compared the selected upregulated miRNAs against downregulated proteins, and selected downregulated miRNAs against upregulated proteins, to identify biologically meaningful regulatory relationships supported by experimental evidence (miRTarBase) or high-confidence predictions (miRbase). These interactions comprise a combination of experimentally validated miRNA–target pairs and high-confidence predicted interactions and therefore represent potential regulatory relationships rather than direct mechanistic proof.

By this approach, we identified 103 downregulated proteins potentially regulated by the 5 selected upregulated miRNAs. Notably, hsa-miR-124-3p and hsa-miR-1-3p act as hub regulators by targeting multiple proteins across several pathways (Figure 4B). Many of these interactions converged on structural and signaling nodes that integrate signals from inflammation, apoptosis, immune evasion, and epithelial integrity, with SQSTM1, CANX, STAT3, IRF9, ITGA2, ITGA3, ITGA5, JUP and MYOF as representative targets. Their reduced expression may impair antiviral and inflammatory signaling, weaken proteostatic and autophagic control, and destabilize cell–matrix interactions, potentially compromising host stress responses and tissue integrity during viral infection.

Among inflammatory mediators, PTGES, LGALS3, GRN, TRIM38 and SQSTM1 were reduced, indicating disruption of both pro- and anti-inflammatory regulation. Survival factors including STAT3, CANX, POMP and NME4 were suppressed, favoring apoptotic susceptibility under stress. Immune defense was weakened through downregulation of IRF9, CANX, CD59, RAB7A, EEA1 and LAMP1/2, suggesting impaired interferon responses and antigen presentation. Structural and adhesion proteins, including keratins, JUP, ITGA2, ITGA3, ITGA5 and MYOF, were also diminished, compromising epithelial cohesion and repair. These coordinated changes illustrate how ORF8-modulated miRNA remodeling reprograms host cells by disturbing inflammatory homeostasis, promoting apoptosis, evading immune surveillance, and weakening epithelial integrity in a manner consistent with cellular states associated with viral infection.

**Figure 5:**
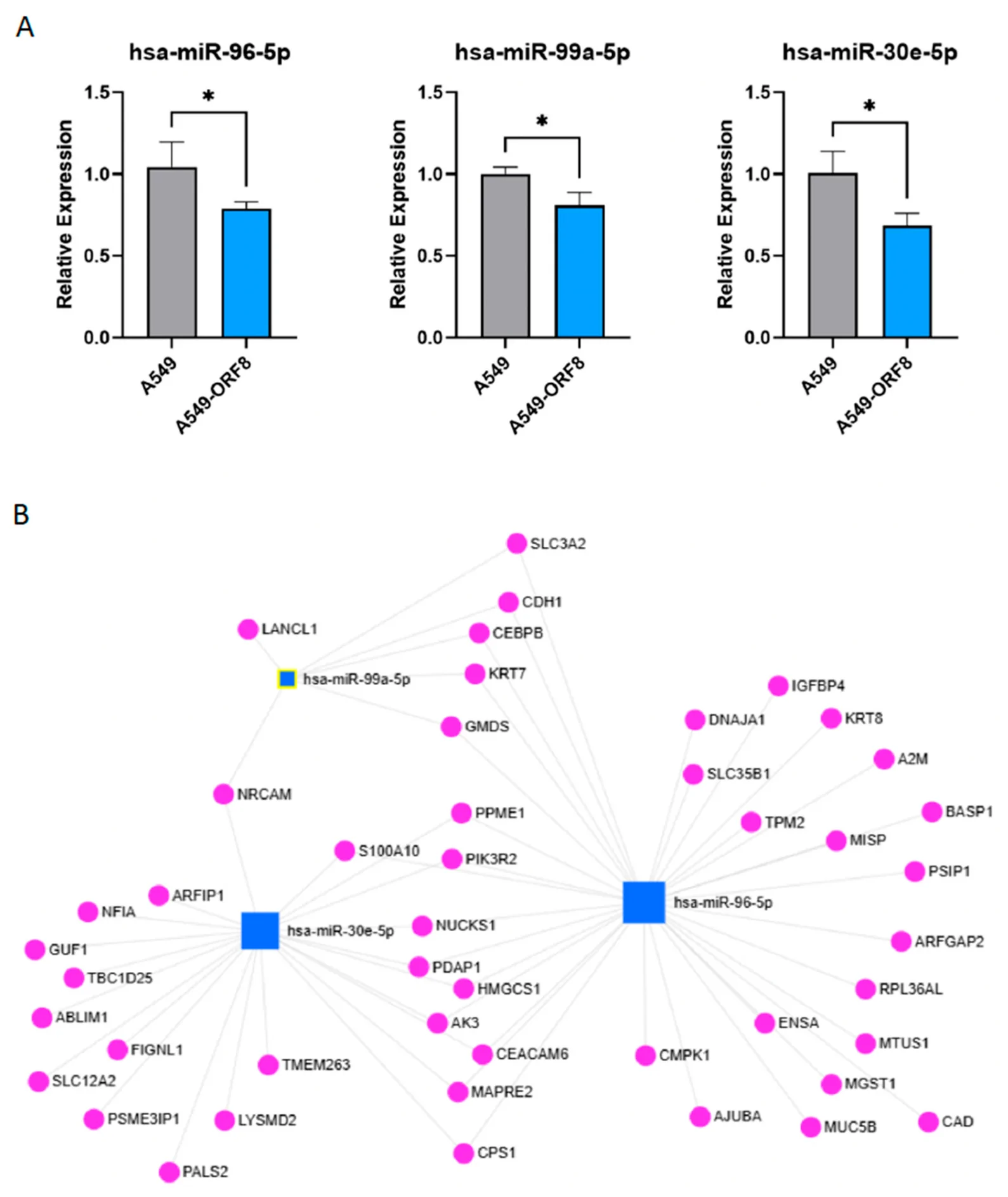
Validation and network-based integration of ORF8-downregulated miRNAs. (**A**). RT–qPCR validation of selected miRNAs significantly downregulated in ORF8-transduced A549 cells compared with control cells. Relative expression levels are shown for hsa-miR-96-5p, hsa-miR-99a-5p, and hsa-miR-30e-5p. Data are expressed as mean ± SEM from 3 independent experiments and are normalized to endogenous controls. Statistical analysis was performed using an unpaired two-tailed Student’s *t*-test (* *p* < 0.05). (**B**). Network representation of ORF8-downregulated miRNAs and their predicted or experimentally supported protein targets. Upregulated miRNAs are shown in blue, and protein targets are shown in pink.

In parallel, we validated selected miRNAs that were significantly downregulated upon ORF8 expression. RT–qPCR analysis confirmed reduced expression of hsa-miR-96-5p, hsa-miR-99a-5p and hsa-miR-30e-5p in ORF8-transduced cells (Figure 5A). These miRNAs have previously been associated with cellular stress responses, metabolic regulation and antiviral signaling [48].

Network analysis of ORF8-downregulated miRNAs and their predicted protein targets revealed 46 upregulated proteins as potential targets of the 3 selected downregulated miRNAs (Figure 5B). hsa-miR-96-5p and hsa-miR30e-5p appeared as the most prominent regulators, with networks extending over proteins involved in stress response, adhesion, and cytoskeletal organization. Several upregulated proteins identified as potential targets of the selected downregulated miRNAs are functionally linked to cell survival, protein homeostasis and resistance to stress-induced apoptosis. These included DNAJA1, a co-chaperone involved in protein folding and quality control, CAD, a key enzyme in de novo pyrimidine biosynthesis, and MGST1, which participates in cellular defense against oxidative stress. Additional upregulated proteins such as AK3, CEBPB and S100A10 are associated with metabolic adaptation, inflammatory regulation and membrane dynamics. The increased abundance of these proteins suggests that downregulation of specific miRNAs may contribute to enhanced proteostatic capacity, metabolic support and stress resilience in ORF8-transduced cells.

The complementary nature of upregulated and downregulated miRNA networks suggests that ORF8 reshapes host gene expression through both activation and repression of distinct post-transcriptional regulatory circuits.

### 2.6. JUP Is a Structural Target of ORF8-Modulated miRNA Networks

Among the proteins identified through integrative miRNA–proteome analysis, junction plakoglobin (JUP) emerged as a representative structural protein targeted by multiple ORF8-modulated miRNAs. JUP plays a critical role in adherens junctions and epithelial integrity, processes that are frequently disrupted during viral infection and inflammation [49].

**Figure 6:**
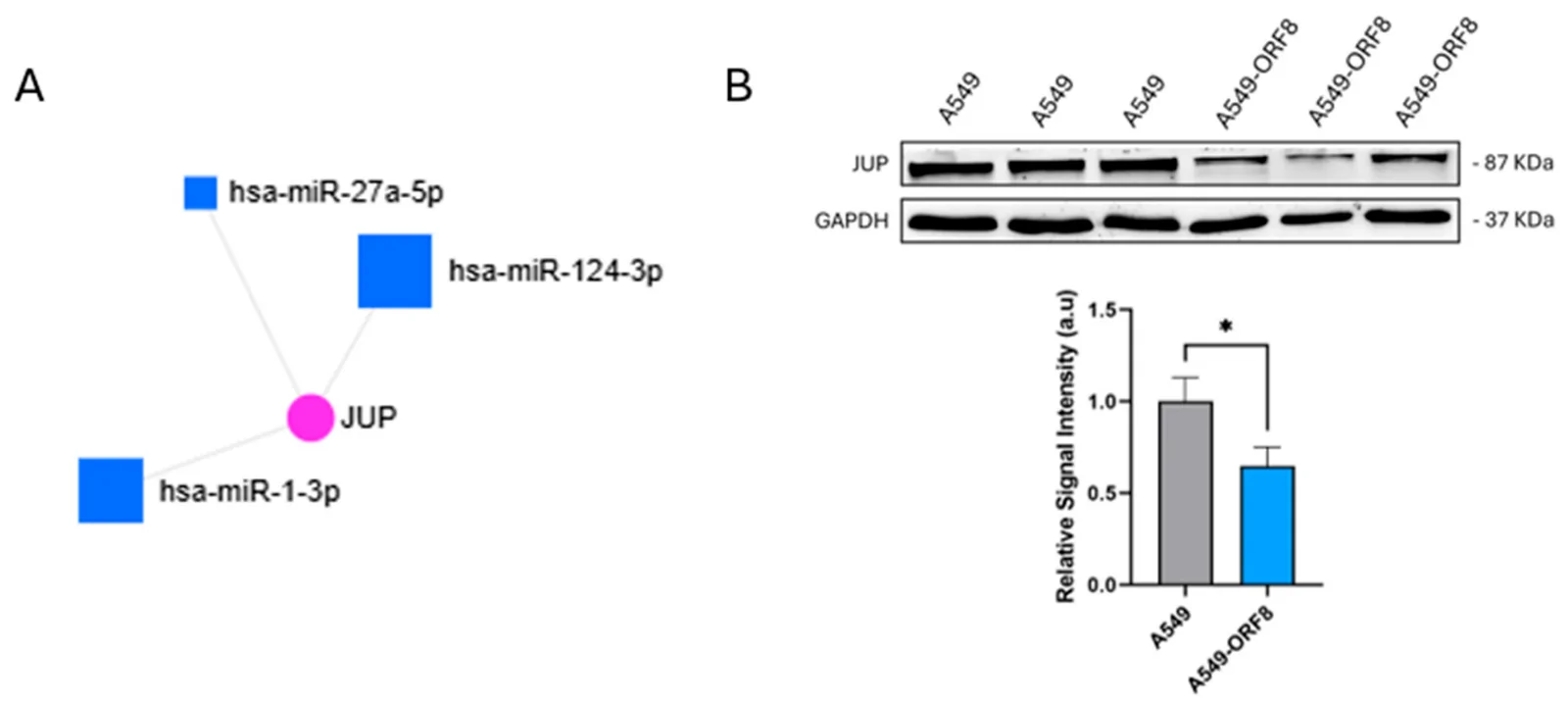
JUP is a representative structural target of ORF8-modulated miRNAs. (**A**). Network representation of ORF8-modulated miRNAs predicted to regulate junction plakoglobin (JUP). miRNAs are shown as nodes connected to JUP, illustrating the convergent post-transcriptional regulation of this structural protein. (**B**). Western blot analysis showing reduced JUP protein levels in ORF8-transduced A549 cells compared with control cells. Densitometric quantification of JUP protein levels is normalized to the loading control GAPDH. Statistical analysis was performed using an unpaired two-tailed Student’s *t*-test (* *p* < 0.05).

Network analysis revealed convergent regulation of JUP by several ORF8-modulated miRNAs (Figure 6A), suggesting coordinated post-transcriptional control. Importantly, Western blot analysis confirmed a significant reduction in JUP protein levels in ORF8-transduced cells compared with control cells (Figure 6B). This experimental validation supports the notion that ORF8-associated miRNA remodeling may contribute to measurable alterations in epithelial structural components, potentially contributing to barrier dysfunction and altered cell–cell adhesion during infection.

Taken together, these analyses reveal a hub-like regulatory architecture in which ORF8-modulated miRNAs target multiple proteins across interconnected pathways involved in inflammation, apoptosis, immune regulation, epithelial integrity and autophagy. Rather than isolated regulatory events, these patterns indicate coordinated remodeling of host cellular networks, with individual miRNAs acting as central nodes controlling clusters of functionally related proteins. To complement these molecular findings with a functional readout of ORF8 activity, we next validated the pro-inflammatory phenotype associated with ORF8 expression in our cellular model. ORF8-transduced cells exhibited significantly increased IL-6 mRNA and secreted protein levels compared with control cells (Figure S1A,B). This response was reduced by dexamethasone treatment without affecting cell viability (Figure S1C,D), confirming both the biological activity of the ORF8 construct and the pharmacological sensitivity of this inflammatory output.

Together with the miRNA and proteomic data presented above, these findings illustrate how ORF8-associated regulatory perturbations may converge on multiple downstream phenotypes, including inflammation, while acting through broader post-transcriptional networks.

## 3. Discussion

In this study, we demonstrate that expression of the SARS-CoV-2 accessory protein ORF8 is associated with a pro-inflammatory phenotype in lung epithelial cells and with extensive remodeling of host miRNA networks and functionally coherent proteomic alterations. By integrating small RNA sequencing, quantitative proteomics and targeted experimental validation, we provide evidence that ORF8-associated host reprogramming operates, at least in part, through miRNA-mediated post-transcriptional regulation. Rather than representing isolated molecular events, our findings support a network-based model linking inflammatory signaling, epithelial integrity, intracellular trafficking, stress responses and metabolic adaptation. These results expand current understanding of ORF8 biology and suggest that modulation of host regulatory RNA networks may contribute to several pathogenic processes associated with SARS-CoV-2 infection. Importantly, our data indicate that ORF8-driven inflammation may arise not only from direct effects on cytokine signaling but also from broader miRNA-mediated perturbations affecting cellular homeostasis and tissue integrity.

A key finding of this work is the extensive reshaping of the miRNA landscape induced by ORF8 expression (Figure 1A; Table 1). Both upregulated and downregulated miRNAs were identified, indicating that ORF8 does not globally suppress host miRNA biogenesis but instead promotes structured redistribution of post-transcriptional regulators. Similar large-scale miRNA remodeling has been described for other viral infections and is thought to represent an efficient mechanism to manipulate host gene expression while minimizing direct viral–host protein interactions [15,48]. Several of the most strongly modulated miRNAs identified here, including hsa-miR-1-3p, hsa-miR-124-3p, hsa-miR-145-5p and hsa-miR-27a-5p, have previously been implicated in immune regulation, inflammatory signaling and epithelial homeostasis [24,25,28,50], suggesting that ORF8 exploits pre-existing regulatory circuits rather than inducing entirely novel responses. While the magnitude of individual miRNA alterations is modest, such shifts are consistent with the fine-tuning regulatory role typical of miRNA biology. Rather than relying on single, high-magnitude changes, the biological impact of ORF8 likely stems from the coordinated remodeling of broader miRNA-target networks, where cumulative minor variations across multiple nodes can drive substantial functional outcomes.

An important question raised by these findings is the precise mechanism by which ORF8 drives this widespread miRNA remodeling. This landscape alteration could occur through several non-mutually exclusive pathways. ORF8 may modulate the host transcriptional program, thereby altering the expression of primary miRNA transcripts or their key transcription factors. Alternatively, it could directly interact with or disrupt components of the core miRNA biogenesis machinery, such as the Microprocessor complex (Drosha/DGCR8), the nuclear export factor Exportin-5, or cytoplasmic Dicer. Finally, potential effects on mature miRNA stability cannot be ruled out, and future biochemical studies will be required to distinguish among these possibilities.

Functional enrichment analysis of predicted miRNA target genes revealed convergence on pathways related to protein processing in the endoplasmic reticulum, cytoskeletal organization, focal adhesion, endocytosis and innate immune signaling (Figure 1B). These pathways are highly relevant to SARS-CoV-2 infection and are consistent with previous reports describing ORF8 as a modulator of ER-associated processes and intracellular trafficking [7,11]. Moreover, enrichment of MAPK, mTOR, TGF-β and RIG-I-like receptor signaling pathways aligns with transcriptomic and proteomic studies of SARS-CoV-2-infected cells and patient samples, which have highlighted dysregulation of these signaling axes as central features of COVID-19 pathogenesis [1,23,51].

Integration of miRNA and proteomic datasets further strengthened this model. DEGs in ORF8-transduced cells were not randomly distributed but segregated into biologically coherent functional categories (Figure 3). Upregulated proteins were enriched in immune defense, lysosomal function and viral life cycle-associated processes, whereas downregulated proteins were associated with cytoskeletal organization, metabolic adaptation and stress response pathways. This stratification mirrors the functional predictions derived from miRNA target analysis and supports the concept that miRNA remodeling can drive coordinated protein-level changes, as previously described in other viral systems [15,22]. Notably, several of the affected pathways are intimately connected with inflammatory regulation, suggesting that ORF8-induced miRNA dysregulation may contribute to inflammation both directly, through modulation of immune signaling, and indirectly, through perturbation of structural and metabolic homeostasis.

Experimental validation of selected modulated miRNAs confirmed the robustness of the sequencing data (Figure 4A and Figure 5A). Network analysis revealed that these miRNAs function as hub regulators, targeting multiple proteins across interconnected pathways (Figure 4B and Figure 5B). Such hub-based regulatory architectures are characteristic of miRNA-mediated control and enable relatively small changes in miRNA abundance to exert broad effects on host gene expression [52]. In the context of viral infection, this strategy may represent an efficient mechanism to reprogram host cells while minimizing the need for extensive viral protein–host interactions [15]. Importantly, several of the validated miRNAs identified in this study, including hsa-miR-1-3p, hsa-miR-27a-3p and hsa-miR-96-5p, have also been reported to be dysregulated in COVID-19 patient samples, displaying concordant directions of regulation (Figure 2) [26,44,45]. Specifically, hsa-miR-1-3p and hsa-27a-3p were consistently upregulated, whereas hsa-miR-96-5p was downregulated both in our experimental system and in patient-derived datasets. Notably, hsa-miR-1-3p has been associated with disease severity in hospitalized COVID-19 patients [26], suggesting that some components of the ORF8-induced miRNA signature may reflect clinically relevant host responses. While these observations do not establish a direct causal link between ORF8 expression and patient-derived miRNA profiles, they raise the possibility that ORF8 contributes to shaping miRNA signatures observed during SARS-CoV-2 infection.

Among the proteins identified through integrative analysis, junction plakoglobin (JUP) emerged as a representative structural target regulated by multiple ORF8-modulated miRNAs. JUP is a key component of adherens junctions and desmosomes and plays a critical role in maintaining epithelial integrity and cell–cell adhesion [49]. Disruption of junctional complexes has been linked to epithelial barrier dysfunction and inflammatory signaling during viral infection, including SARS-CoV-2 [20,46,53,54]. Network analysis revealed convergent miRNA regulation of JUP (Figure 6A), and Western blot validation confirmed a significant reduction in JUP protein levels upon ORF8 expression (Figure 6B). This finding provides a supporting example of how ORF8-associated miRNA remodeling may contribute to structural alterations with potential consequences for epithelial barrier function and tissue homeostasis. More broadly, our data support a model in which ORF8-driven miRNA dysregulation coordinately affects epithelial integrity, cytoskeletal organization and metabolic adaptation, thereby generating a cellular environment that may further amplify or sustain inflammatory responses. These findings extend current understanding of ORF8 function beyond previously described mechanisms such as MHC-I downregulation and cytokine modulation [5,8,10]. While earlier studies have focused on transcriptional and protein–protein interaction-based effects of ORF8, our data identify miRNA-mediated post-transcriptional regulation as an additional and complementary layer of host manipulation. This mechanism may help explain how ORF8 simultaneously influences immune signaling, stress responses and epithelial organization.

From a translational perspective, miRNA networks represent an attractive interface for viral host manipulation due to their capacity for signal amplification and pathway integration. Several of the miRNAs identified here have previously been detected in respiratory samples or circulating extracellular vesicles during viral infections, including SARS-CoV-2 [18,26,44,45]. ORF8-associated miRNA signatures could therefore serve as biomarkers of viral protein activity or host response dysregulation. However, the pleiotropic nature of miRNA regulation necessitates careful consideration of therapeutic targeting strategies.

Several limitations should be acknowledged. First, the use of ectopic ORF8 expression in A549 cells, a transformed lung epithelial line, does not fully recapitulate the physiological context of SARS-CoV-2 infection, including viral replication dynamics, the presence of other viral proteins, or the complex microenvironment of the respiratory tract. Furthermore, our ectopic expression system does not replicate the dynamic kinetics or absolute protein levels of ORF8 typical of a natural SARS-CoV-2 infection. While this approach allows for a controlled, mechanistic dissection of how ORF8 modulates miRNA networks, validation of selected miRNA changes using full-length SARS-CoV-2 infection will be required to determine whether these effects are reproduced during the complete viral replication cycle and are not attributable to ORF8 over-expression. Second, although integrative analyses strongly suggest functional miRNA–protein relationships, direct validation of individual miRNA–target interactions (e.g., by luciferase reporter assays or CRISPR-based approaches) was beyond the scope of this work. Third, our network analysis relies on predicted miRNA targets and inverse correlations, which may include false-positive interactions; experimental perturbation of candidate miRNAs is required to confirm their regulatory roles. Finally, the extent to which ORF8 alone accounts for the miRNA changes observed in COVID-19 patients remains unknown, as other viral and host factors likely contribute. In addition, this study used the Wuhan-Hu-1 ORF8 sequence, whereas ORF8 is highly variable among SARS-CoV-2 lineages, including deletions and stop-codon mutations. Therefore, our findings may not be uniformly applicable to variants with altered or absent ORF8, and further studies using representative ORF8 variants will be required to determine the conservation of these miRNA regulatory effects.

In conclusion, these findings expand the functional repertoire of ORF8 beyond its established roles in immune evasion, suggesting that it may also act as a modulator of host post-transcriptional regulatory networks. By integrating miRNA profiling, proteomic analysis and experimental validation, we reveal a network-based mechanism through which ORF8 may contribute to immune modulation, stress adaptation and epithelial structural alterations during SARS-CoV-2 infection. Collectively, our results support the concept that ORF8-induced miRNA remodeling represents a central regulatory layer linking inflammatory signaling, epithelial integrity and cellular reprogramming, thereby potentially contributing to multiple aspects of COVID-19 pathogenesis.

## 4. Materials and Methods

### 4.1. Lentivirus Production, Cell Culture and Transduction

ORF8 coding sequences (codon-optimized for mammalian expression) were cloned into the pLVX-EF1α-IRES-Puro lentiviral expression vector (Clontech, Takara, Mountain View, CA, USA) to generate constructs encoding the SARS-CoV-2 ORF8 accessory protein (Wuhan-Hu-1 isolate, Wuhan, China). Pseudotyped lentiviral particles were produced at the Viral Vector Unit (ViVU) of the Centro Nacional de Investigaciones Cardiovasculares (CNIC) by co-transfecting HEK293T cells with the pLVX-ORF8 plasmid, pCMV-Gag-Pol and pCMV-VSV-G using Lipofectamine 2000 (Thermo Fisher Scientific, Waltham, MA, USA), according to the manufacturer’s instructions. Supernatants were collected 48 h post-transfection, centrifuged at 500× *g* for 10 min to remove cell debris, concentrated using Amicon Ultra-15 centrifugal filter units, and titrated by serial dilution. A549 cells were selected as a well-established and highly tractable model for studying lung epithelial cell biology and host–pathogen interactions. Their high transduction efficiency and robust growth properties make them ideal for generating stable, homogeneous cell populations for controlled mechanistic and multi-omics studies. A549 pulmonary epithelial cells (ATCC CRM-CCL-185) were transduced with ORF8 lentivirus at a multiplicity of infection (MOI) of 10 for 24 h, followed by puromycin selection (2 µg/mL) following the experimental procedure and expression characterization described previously [20,46].

### 4.2. RNA Extraction and Sequencing

Control A549 and A549-ORF8 cells were seeded in 6-well plates and lysed using TRIzol reagent (Thermo Fisher Scientific) for total RNA isolation. All samples were prepared in biological triplicates. RNA extraction was performed according to the manufacturer’s protocol, and RNA concentration was measured using a Nanodrop 1000 spectrophotometer (Thermo Fisher Scientific). RNA integrity was assessed using an Agilent Bioanalyzer (Agilent Technologies, Santa Clara, CA, USA), and only samples with RNA integrity number (RIN) values above 9.90 were used for sequencing.

Small RNA library preparation and sequencing were performed by Novogene (Beijing, China). Briefly, 1 µg of total RNA per sample was used for small RNA library construction using the NEBNext Multiplex Small RNA Library Prep Set for Illumina (New England Biolabs, Ipswich, MA, USA), following the manufacturer’s instructions. Adapters were ligated to the 3′ and 5′ ends of small RNAs, followed by reverse transcription and PCR amplification. Amplified libraries were size-selected to enrich for fragments corresponding to small RNAs (18–30 nt), and library quality and concentration were assessed prior to sequencing. Sequencing was performed on an Illumina platform in single-end mode, generating high-quality reads for downstream bioinformatic analysis.

### 4.3. Small RNA Sequencing Data Processing and Differential Expression Analysis

Raw sequencing reads were first subjected to quality control and adapter trimming. Clean reads were mapped to the human reference genome using Bowtie (v0.12.9) with the parameters “-v 0 -k 1” to allow only perfect matches and retain uniquely mapped reads. Known miRNAs were identified by alignment to miRBase, and novel miRNA prediction was performed using a combination of miREvo (v1.1) and miRDeep2 (v0.0.5), integrating ViennaRNA (v2.1.1) for secondary structure prediction. Repeat sequences were identified and filtered using RepeatMasker (v4.0.3) based on RepBase 18.07, including tandem repeat finder (trf) and inverted repeat finder (irf). Quantification of known miRNAs was performed using the quantifier module of miRDeep2 with default parameters.

Differential expression analysis between control and ORF8-transduced A549 cells was conducted using the DESeq2 package (v1.12.0) in R (v3.3.0), which is specifically designed for biological replicates. miRNAs with an adjusted *p*-value (Benjamini–Hochberg correction) < 0.05 were considered significantly differentially expressed. Log2 fold change values were calculated to determine the direction and magnitude of expression changes. Only miRNAs showing consistent expression across biological replicates and meeting statistical significance thresholds were retained for downstream functional and integrative analyses.

### 4.4. Reverse Transcription-Quantitative Real-Time PCR (RT-qPCR)

Total RNA from cell cultures was extracted using TRIzol reagent (Thermo Fisher Scientific) following the manufacturer’s protocol. For miRNA expression analysis, 100 ng of total RNA per sample was reverse-transcribed into cDNA as previously described [55]. PCR reaction mixtures and cycling conditions were performed as reported in [56], and primer sequences are listed in Supplementary Table S2.

To assess the impact of ORF8 expression on pro-inflammatory signaling, *IL-6* mRNA levels were quantified by RT–qPCR in A549-ORF8 and control cells. Total RNA was extracted as described above and reverse-transcribed into cDNA. *IL6* expression levels were determined using gene-specific primers and normalized to endogenous controls (*GAPDH*) using the 2^−ΔΔCt^ method. For pharmacological modulation, cells were treated with dexamethasone (50 or 100 µM) for 24 h prior to RNA extraction. Relative *IL6* expression levels were compared between untreated and dexamethasone-treated conditions in both control and ORF8-expressing cells.

Relative miRNA expression levels were calculated using the 2^−ΔΔCt^ method [57] with GenEx software (GenEx 6, bioMCC, Freising, Germany). Statistical analyses and graphical representations were performed using GraphPad Prism software (v9.4.1). Comparisons between groups were conducted using unpaired Student’s *t*-test or one-way analysis of variance (ANOVA) with Tukey’s multiple comparisons test, with *p* < 0.05 considered statistically significant.

### 4.5. ELISA Assay

Cells were seeded (6 × 10^4^) in 24-well plates with 1 mL of medium, and cell supernatants were collected after 24 h. Interleukin levels were detected with a Human IL-6 Uncoated ELISA Kit (Invitrogen, Waltham, MA, USA, #88-7066-88) according to the manufacturer’s instructions.

### 4.6. Cell Viability

A549 cells expressing control or ORF8 proteins were seeded in 96-well plates at densities of 1 × 10^4^. Cells were treated with 50 or 100 µM dexamethasone for 24 h. Cell viability was subsequently assessed using the MTT [3-(4,5-dimethylthiazol-2-yl)-2,5-diphenyltetrazolium bromide] assay. Briefly, cells were incubated with MTT reagent (Sigma-Aldrich, St. Louis, MO, USA, #M2128) at a final concentration of 500 μg/mL for 2 h at 37 °C. After incubation, the medium was removed and the resulting formazan crystals were solubilized in DMSO. Absorbance was measured at 570 nm using a Varioskan LUXmicroplate reader(Thermo Ficher Scientific).

### 4.7. Sample Preparation for Proteomics

ORF8-A549 cells (5 × 10^6^ cells per biological replicate) were lysed with 200 µL of 5% sodium dodecyl sulfate (SDS) and 25 mM triethylammonium bicarbonate (TEAB) supplemented with 10 mM tris(2-carboxyethyl) phosphine (TCEP) and 10 mM chloroacetamide (CAA). The lysates were sonicated using an ultrasonic processor UP50H (Hielscher Ultrasonics, Teltow, Germany) for 1 min on ice (0.5 cycles, 100% amplitude) and incubated at 60 °C for 60 min. The protein extracts were centrifuged at 18,400× *g* for 10 min and the supernatant was transferred to a new tube. The total protein was quantified by PIERCE 660 nm reagent (Thermo Scientific) supplemented with ionic Detergent Compatibility Reagent (Thermo Scientific). Protein digestion on S-Trap columns (Protifi, Fairport, NY, USA) was performed following the manufacturer’s instructions with minor changes, as previously referred to [58]. Briefly, 80 µg of protein from each sample was digested at 37 °C overnight using a trypsin:protein ratio of 1:15. Tryptic peptides were quantified by fluorimetry (QuBit, Thermo Fisher Scientific), according to the manufacturer’s instructions, and labeled using Tandem Mass Tags (TMT)pro™ 16plex kit (Thermo Fisher Scientific). Briefly, 25 µg of tryptic peptides from each sample was resuspended in 168 mM EPPS buffer and 16% ACN and labeled for 2h with distinct TMT label reagents. A pool of all digested samples (1.7 µg/each sample) to be used as internal standard (IS) in the experiment was labeled with 134N TMT tag. The labeling reaction was stopped with 0.3% hydroxylamine and the samples were combined in equal parts.

### 4.8. Basic-pH Fractionation Using SDB-RPS STAGE Tips

The fractionation of TMT-labeled peptides was performed using in-house-made STAGE tips prepared from SDB-RPS solid-phase extraction disks (Empore™, Oxford, PA, USA). The STAGE tip was prepared by cutting 12 pieces of SDB-RPS solid-phase extraction disks with a 16-gauge blunt-end needle and packing them into a 200 µL tip, as described previously [59]. The prepared tip was inserted into the top of a 2-mL tube using an adapter made at home and used as a fractionation column. TMT-labeled peptides (80 µg) were resuspended in 100 µL 1% FA (pH < 3), loaded, and fractionated as previously reported [60,61]. The fractionation procedure was carried out similarly to the previously described, but in our case 10 fractions were obtained using a 10-step-wise elution with 100 µL of 5 mM ammonium formate buffer and increasing acetonitrile concentrations (0, 5.0%, 7.5%, 10.0%, 12.5%, 15.0%, 17.5%, 20%, 25%, and 45%). Fractions were dried in a speed vacuum and frozen until further processing.

### 4.9. Analysis by Liquid Chromatography Coupled to Mass Spectrometry OE-240

Each sample was quantified by fluorimetry (Qubit) and 1 µg was individually analyzed by nano-Liquid Chromatography coupled to Electrospray Ionization Tandem Mass Spectrometry (nanoLC-ESI-MS/MS) analysis using an Ultimate 3000 nano HPLC system (Thermo Fisher Scientific) coupled online to an Orbitrap Exploris™ 240 mass spectrometer (Thermo Fisher Scientific). Each sample (1 µg in 5 µL of sample resuspended in mobile phase A) were loaded on a 50 cm  ×  75 μm Easy-spray PepMap C18 analytical column (Thermo Fisher Scientific) at 45 °C and were separated at a flow rate of 300 nL/min using a 120 min gradient ranging from 2% to 95% mobile phase B (mobile phase A: 0.1% formic acid (FA); mobile phase B: 80% acetonitrile (ACN) in 0.1% FA). To avoid carry-over, two 40 min blank samples (mobile phase A) were systematically run between samples. Data acquisition was performed using a data-dependent top method, in full scan positive mode, scanning 375 to 1200 *m*/*z*. MS1scans were acquired at an orbitrap resolution of 60,000 at *m*/*z* 200, with a normalized automatic gain control (AGC) target of 300%, a radio frequency (RF) lens of 80%, and an automatic maximum injection time (IT). The top 20 most intense ions from each MS1 scan were selected and fragmented with a Higher-energy collisional dissociation (HCD) of 34%. Resolution for HCD spectra was set to 45,000 at *m*/*z* 200, with an AGC target of 100% and an automatic maximum IT. Isolation of precursors was performed with an isolation window of 0.7 *m*/*z* and 45 s of exclusion duration. Precursor ions with single, unassigned, or six or higher charge states from fragmentation selection were excluded.

### 4.10. Proteomics Data Analysis

Mass spectrometry data were analyzed with Proteome Discoverer (v2.5.0.400) using four search engines (Mascot (v2.7.0), MsAmanda (v2.4.0), MsFragger (v3.1.1), and Sequest HT) using a target/decoy Homo sapiens + SARS-CoV2 Uniprot Knowledgebase database (25 February 2021, 20,462 sequences) with the most common laboratory contaminants (cRAP database with 69 sequences). Search parameters were set as follows: cysteine carbamidomethyl (+57.021464 Da) and TMTpro (+304.207146 Da) on lysine and N-term as fixed modifications; methionine oxidation (M) (+15.994915 Da), N-term acetylation (+42.010565 Da), and Gln→pyro-Glu (−17.026549 Da) as variable modifications. The false discovery rate (FDR) for proteins, peptides, and peptide spectral matches (PSMs) was kept at 1%.

The quantitation was also performed in Proteome Discoverer using the “Reporter Ions Quantifier” feature in the quantification workflow using the following parameters: unique + razor peptides were used for quantitation, co-isolation threshold was set at 50%, signal to noise of reporter ions was 10, and the normalization and scaling were performed considering the total peptide amount and the control (IS) average, respectively. The protein ratio was calculated considering the protein abundance and the hypothesis test was based on a *t*-test (background-based). Protein groups (master proteins) with an FDR lower than 1% and with abundance values in both IS were considered for quantitation. A *p*-value ≤ 0.05 adjusted using Benjamini–Hochberg was set to determine the proteins found differentially expressed in ORF8 compared with the controls. Volcano plot and Principal Component Analysis (PCA) were performed in Proteome Discover considering the differentially expressed proteins in each comparison.

### 4.11. Western Blot

Transduced cells were harvested and lysed in ice-cold RIPA buffer supplemented with complete protease and phosphatase inhibitor cocktails (Sigma-Aldrich) for 30 min at 4 °C. Lysates were cleared by centrifugation and protein concentrations were determined using standard BCA assay. Thirty µg of proteins was mixed with 5× SDS-PAGE sample loading buffer (Nzytech, Lisbon, Portugal, MB11701) and heated at 95 °C for 5 min. Proteins were separated by SDS–polyacrylamide gel electrophoresis and transferred onto nitrocellulose membranes using a Mini Trans-Blot system (Bio-Rad, Hercules, CA, USA). Membranes were blocked for 1 h with 5% BSA in Tris-buffered saline containing Tween-20 and incubated with the primary mouse anti-γ-catenin (Santa Cruz Biotechnology, Santa Cruz, CA, USA, Cat#sc-514115) and secondary antibodies StarBright Blue 700 anti-mouse (Bio-Rad, Cat# 12004159). Protein bands were visualized by fluorescence using a ChemiDoc Imaging System (Bio-Rad). Relative protein expression levels were quantified by densitometry and normalized to the loading control GAPDH (Sigma Aldrich, Cat#G8795).

### 4.12. Functional Enrichment Analysis

Functional annotation of differentially expressed miRNAs and proteins was performed to identify biological processes and pathways potentially affected by ORF8 expression. For miRNA analysis, significantly upregulated and downregulated miRNAs were analyzed separately. Predicted and experimentally validated target genes were retrieved using miRNet (https://www.mirnet.ca), integrating information from miRTarBase v9.0 and miRbase v9.0. To increase biological relevance and reduce false-positive interactions, we retained only interactions supported by at least two databases or with high-confidence scores (miRDB score > 90). To further refine these interactions, we calculated Spearman correlation coefficients between the normalized expression values of each miRNA and its predicted target proteins across the three biological replicates of control and ORF8-transduced cells, using matched samples for both RNA-seq and proteomics. Only inverse correlations (upregulated miRNA with downregulated protein, and vice versa) with a coefficient |r| > 0.6 were retained for network construction. Specifically, upregulated miRNAs were intersected with downregulated proteins, and downregulated miRNAs were intersected with upregulated proteins, following the principle of inverse miRNA–target regulation.

For proteomic data, differentially expressed proteins identified in ORF8-transduced versus control A549 cells were stratified into upregulated and downregulated groups and analyzed separately. Functional enrichment analysis was performed using the STRING database (version 12.0; https://string-db.org). Enrichment was assessed for GO biological processes and KEGG pathways. Enrichment results were visualized as bubble plots, in which bubble size represents the number of proteins associated with each term and colour indicates statistical significance (FDR). This approach enabled identification of coordinated functional modules affected by ORF8 expression without assuming direct causal interactions.

## Figures and Tables

**Table 1:** Top 30 ORF8-modulated miRNAs identified by small RNA sequencing. The table shows the most strongly modulated miRNAs in ORF8-transduced A549 cells compared with control cells, ranked by Manhattan distance.

miRNA	Change	Fold Change (log2)	Significance	Manhattan Distance
hsa-miR-4684-3p	Increased	2.5897	19.4367	22.0264
hsa-miR-1-3p	Increased	4.1173	15.3397	19.4570
hsa-miR-184	Increased	5.0154	12.7769	17.7923
hsa-miR-129-5p	Increased	2.3122	14.6564	16.9686
hsa-miR-767-5p	Decreased	−5.2160	8.30377	13.5197
hsa-miR-204-5p	Increased	4.7140	8.80150	13.5155
hsa-miR-514a-3p	Decreased	−5.1821	8.24202	13.4241
hsa-miR-105-5p	Decreased	−4.9717	7.04319	12.0148
hsa-miR-202-5p	Increased	3.0748	7.08673	10.1615
hsa-miR-224-5p	Increased	2.2173	6.88505	9.10235
hsa-miR-30e-5p	Decreased	−1.4330	7.62198	9.05498
hsa-miR-3176	Decreased	−4.2427	4.71974	8.96244
hsa-miR-27a-5p	Increased	1.6184	6.88505	8.50345
hsa-miR-143-3p	Increased	3.2002	4.55025	7.75045
hsa-miR-96-5p	Decreased	−2.1071	5.46935	7.57645
hsa-miR-145-5p	Increased	3.2786	4.01057	7.28917
hsa-miR-451a	Increased	2.0545	5.12676	7.18126
hsa-miR-1246	Increased	2.9533	4.21262	7.16592
hsa-miR-124-3p	Increased	3.5945	3.53299	7.12749
hsa-miR-509-3-5p	Decreased	−3.6737	3.33577	7.00947
hsa-miR-4798-5p	Decreased	−3.6134	3.26705	6.88045
hsa-miR-450a-2-3p	Decreased	−3.5429	3.10496	6.64786
hsa-miR-409-3p	Increased	1.7723	4.76316	6.53546
hsa-miR-500a-5p	Decreased	−3.3351	3.18415	6.51925
hsa-miR-539-5p	Decreased	−3.4781	3.02118	6.49928
hsa-miR-99a-5p	Decreased	−1.6249	4.71974	6.34464
hsa-miR-374b-5p	Decreased	−1.9564	4.35094	6.30734
hsa-miR-374c-3p	Decreased	−1.9564	4.35094	6.30734
hsa-miR-1252-5p	Decreased	−3.3918	2.80815	6.19995
hsa-miR-454-3p	Decreased	−1.6722	4.30603	5.97823

## Data Availability

The small RNA sequencing datasets generated and analyzed during the current study have been deposited in the NCBI Sequence Read Archive (SRA) database under BioProject accession number PRJNA1504941 (BioSample accessions SAMN62071040 to SAMN62071045).

## Supplementary Materials

The following supporting information can be downloaded at: https://www.mdpi.com/article/10.3390/ijms27188363/s1.
