# Supplementary material for: SARS-CoV-2 ORF8 Exploits Host miRNA Networks to Rewire Post-Transcriptional Regulation
Source: Int J Mol Sci. 2026 Sep 19;27(18):8363. doi: 10.3390/ijms27188363 (PMC13607167; doi:10.3390/ijms27188363)
Supplement: Supplementary file 1 [file ijms-27-08363-s001.zip › Supplementary Figures ORF8_V4.pdf]

Figure S1

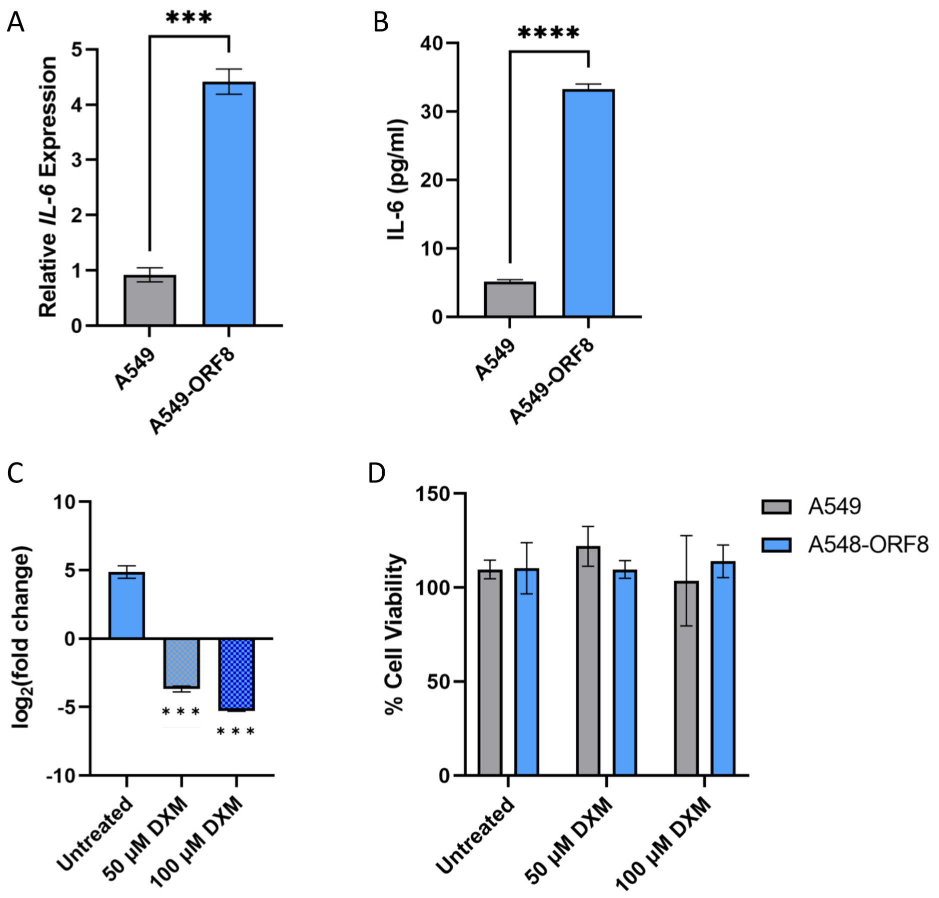

Figure S1. ORF8 expression confers a functionally active phenotype in lung epithelial cells. **A**. Relative *IL-6* mRNA expression levels in A549-ORF8 cells compared with A549 control cells, determined by RT–qPCR. **B**. Secreted IL-6 protein levels in cell culture supernatants measured by ELISA. **C.** Effect of dexamethasone treatment on *IL-6* mRNA expression in ORF8-expressing cells. **D**. Cell viability assessed by MTT assay in A549 control and ORF8-expressing cells. Values are means ± SEM (n=3) per group. Statistical analysis was performed using an unpaired two-tailed Student's t-test. Statistical significance is given as follows: ***p < 0.001 and ****p < 0.0001 to A549 control cells (A,B) or untreated A549-ORF8 cells (C).
